# Metacognitive Beliefs and Suicidal Ideation: An Experience Sampling Study

**DOI:** 10.3390/ijerph182312336

**Published:** 2021-11-24

**Authors:** Vikki Aadahl, Adrian Wells, Robert Hallard, Daniel Pratt

**Affiliations:** 1 Centre for New Treatments and Understanding in Mental Health (CeNTrUM), Division of Psychology & Mental Health, School of Health Sciences, Faculty of Biology, Medicine and Health, University of Manchester, Manchester M13 9PL, UK; adrian.wells@manchester.ac.uk (A.W.); Robert.Hallard@cntw.nhs.uk (R.H.); daniel.pratt@manchester.ac.uk (D.P.); 2 Greater Manchester Mental Health NHS Foundation Trust, Trust Headquarters, Bury New Road, Prestwich, Manchester M25 3BL, UK

**Keywords:** metacognition, S-REF model, cognition, self-harm

## Abstract

The current study aimed to examine the relationship between metacognitive beliefs about suicidal ideation and the content and process of suicidal ideation. This was to examine the potential contribution of the Self-Regulatory Executive Function (S-REF) model (Wells and Matthew, 2015) to suicidal ideation. Twenty-seven participants completed both trait and state-level measures of suicidal ideation, negative affect, defeat, hopelessness, entrapment and metacognitive beliefs. Experience Sampling Methodology (ESM) was adopted to measure state-level measurements with participants invited to complete an online diary up to seven times a day for six days. Multi-level modelling enabled a detailed examination of the relationships between metacognitive beliefs and suicidal ideation. Positive (β = 0.241, *p* < 0.001) and negative (β = 0.167, *p* < 0.001) metacognitive beliefs about suicidal ideation were positively associated with concurrent suicidal ideation even when known cognitive correlates of suicide were controlled for. The results have important clinical implications for the assessment, formulation and treatment of suicidal ideation. Novel meta-cognitive treatments targeting beliefs about suicidal ideation are now indicated. A limited range of characteristics reported by participants affects the generalizability of findings. Future research is recommended to advance understanding of metacognition and suicide but results demonstrate an important contribution of the S-REF model.

## 1. Introduction

Over 700,000 people die each year by suicide [1], so improving our understanding of suicide is imperative [2]. Since death by suicide is almost always preceded by suicidal thoughts, all aspects of suicidal ideation should be considered in research and treatment [3]. Klonsky and May (2015) argue that any model of suicide should explain the development of suicidal ideation and the progression to suicidal behavior [4]. The current study addresses the former.

Kerkhof and van Spijker (2011) argued that the process of suicidal ideation is similar to worry and rumination [5]. Similarly, in the S-REF model, suicidal ideation is seen as a type of rumination that is principally a coping strategy, and this can be distinguished from beliefs about that process or worry about that process, which are higher level metacognitions (since they are cognitions about cognition). Worry and rumination have been conceptualised as cognitive coping strategies that can have paradoxical effects of maintaining emotional difficulties through affecting the processes required for effective self-regulation [6,7]. The Self-Regulatory Executive Function (S-REF) model suggests that worry and rumination are associated with metacognitive beliefs that contribute to the persistence of these processes [6,7]. Such beliefs concern the usefulness of worry/rumination (e.g., “Analysing my feelings will help me get better”) and beliefs concerning the uncontrollability and threat related to thoughts (e.g., “I can’t stop thinking about harming myself”). Whilst this model has gained status in explaining anxiety [8] and depression [9,10,11], it has not previously been applied to suicidal ideation. However, to the extent that suicidal ideation may represent a form of worry and rumination, it should draw on the same set of underlying metacognitions described in this model.

The S-REF model [6,7] is a metacognitive, information processing model. It proposes that a ‘cognitive-attentional syndrome (CAS)’ consisting of heightened self-focus, repetitive and difficult to control negative thinking (worry and rumination), maladaptive coping behaviour and threat monitoring contribute to the maintenance of psychological difficulties. The engagement of the CAS is influenced by metacognition, which is described as the monitoring, evaluating and regulating of cognition [12,13]. Metacognition incorporates both metacognitive knowledge and metacognitive regulation. Metacognitive knowledge is information individuals have about their own thinking (including metacognitive beliefs). Metacognitive regulation refers to the strategies used to change thinking.

In the S-REF model, positive (e.g., “I must worry in order to be prepared”) and negative (e.g., “I cannot control my thoughts”) metacognitive beliefs have been distinguished and their influences on the CAS have been elucidated [13] with negative metacognitions posited as the more important influence. Higher levels of negative metacognitive beliefs have been associated with anxiety [14], depression [9] and obsessive compulsive disorder [15]. Emerging evidence suggests that metacognitive beliefs may be a general vulnerability factor for psychological distress, regardless of psychiatric diagnosis [16,17]. Metacognitive beliefs have been found, in prospective cohort studies, to predict subsequent anxiety and depression [11,18], and to mediate the relationship between symptoms of psychological disorders and the associated distress [19].

In addition to more general meta-cognitive beliefs, specific positive and negative metacognitive beliefs may be held about suicidal ideation [5,20]. Rogers and Joiner (2018) found that rumination about suicidal thoughts related to lifetime suicide attempts over and above other risk factors [21]. Williams, Duggan, Crane and Hepburn (2011) found that individuals try to suppress suicidal thoughts and that the severity of an individual’s suicidal ideation is associated with the level of suppression [22]. On the contrary, Vatne and Naden (2012) found that participants held positive metacognitive beliefs about suicidal ideation—a knowledge that suicide offered an ‘open door as consolation’ such that thoughts about taking one’s own life brought “relief, comfort and courage to endure suffering” [23]. Crane et al. (2014) found a significant positive correlation between comfort taken from suicidal ideation and worst ever suicidal ideation [24]. The authors considered whether the same process in Joiner’s Interpersonal Psychological Theory of Suicide (IPTS) [25], with regards to lethal self-harm, could occur in suicidal ideation. The IPTS model suggests that, through habituation and opponent processes, emotional reactions to deliberate self-harm change from fear to a source of emotional relief. They wondered if the same occurs with suicidal thoughts that in time the thoughts create a source of emotional relief. A limitation of these studies is that a distinction is not made between positive beliefs about the event of suicide (“death by suicide could be a positive thing for me”) and positive beliefs about the process of thinking about suicide (“thinking about suicide is a positive thing for me”). Furthermore, the relative contributions of positive and negative metacognitive beliefs have not been specifically evaluated.

The aim of this study was to examine the relationship between suicidal ideation and metacognitive beliefs about suicidal ideation when known risk factors for suicide (depression, defeat, entrapment and hopelessness) are controlled for. The hypotheses were:

**Hypothesis** **1** **(H1).** *Trait-level measures of suicidal ideation, metacognitive beliefs, defeat, entrapment, depression and hopelessness will all be significantly associated with state level scores of suicidal ideation, metacognitive beliefs, defeat, entrapment, depression and hopelessness (to ‘validate’ ESM diary items).*

**Hypothesis** **2** **(H2).** *State-level scores of negative affect, defeat, entrapment and hopelessness will all be significantly associated with state-level scores of suicidal ideation.*

**Hypothesis** **3** **(H3).** *State-level scores of suicide-specific metacognitive beliefs will be significantly associated with state-level scores of suicidal ideation.*

**Hypothesis** **4** **(H4).** *State-level scores of suicide-specific metacognitive beliefs will remain significantly associated with state-levels of suicidal ideation, when state-levels of negative affect, hopelessness, defeat and entrapment are controlled for.*

## 2. Materials and Methods

### 2.1. Participants

Participants were recruited from both the National Health Service (NHS) (inpatient mental health wards) and a third sector organisations (Lancashire Women’s Centre) providing a primary care mental health service in the North of England. Inclusion criteria were self-reported suicidal ideation in the past two months (participants were asked, ‘have you experienced suicidal thoughts in the last two months?’), 18 years old or over, capacity to provide consent and sufficient comprehension/production of the English language. The exclusion criteria were a primary organic disorder or excessive alcohol or drug use that would affect participation, as reported by the participant or observed by the researcher. All participants were assessed by the researchers as having sufficient mental capacity to allow informed consent, in line with the WHO Good Clinical Practice guidelines and the UK Policy Framework for Health and Social Care Research.

Twenty-seven participants agreed to take part who had a mean age of 34.2 years (range = 18–63, SD = 13.9). Twenty four participants went on to complete the diary measure. See Table 1 for demographic details of all 27 participants:

### 2.2. Measurement

#### 2.2.1. State-Level Experience Sampling Methodology (ESM)

ESM [26] is a structured diary technique that requires in-the-moment assessment of a given phenomenon. It was specifically designed to capture momentary ratings of experiences believed to change over time such as mood, thoughts and behaviours [27]. ESM is recognised as offering heightened ecological validity [28]. Spangenberg, Forkmann and Glaesmer (2015) reviewed the use of ESM in suicidal participants finding good compliance rates and an absence of a reactive effect [29]. A distinction is made between measurements at the trait-level via self-report questionnaires and measurement at the state-level with ESM diary items.

The items measuring cognitive constructs were developed based on Williams’ Cry of Pain model [30,31] and O’Connor’s Integrated Motivational-Volitional model of suicidal behaviour [32,33], which emphasises constructs of defeat, entrapment and hopelessness. Validated trait-level measures of these constructs, including the Beck Hopelessness Scale [34], the Defeat scale [35] and Entrapment scale [35] were used as guides. The S-REF model [6,7] and the Metacognitions Questionnaire-30 (MCQ-30) [36] informed the development of the metacognitive belief items. Initial items were trialled with five pilot participants following the same procedure for the main study. Participant feedback alongside consideration of skewness, variability and item-total correlation statistics resulted in a final set of 35 items (thought control strategies were also assessed in these 35 items but not considered within the current study—a full list of diary items presented in Table S1 of Supplementary Materials). For each variable there was a minimum of two items in the ESM diary (apart from cognitive confidence). A summary of the diary items and corresponding constructs can be seen in Table 2. Each item was scored on a 7 point Likert scale ranging from 1 = ‘not at all’ to 7 = ‘very much’.

#### 2.2.2. Trait-Level Self-Report Questionnaire Measures

The following measures were administered to describe participant characteristics and to validate the state-level items:

1. Beck Depression Inventory–II (BDI-II) [37]. Twenty items that assess the symptoms of depression with excellent test-retest reliability (r = 0.93) [37] and excellent internal reliability in clinical samples (α = 0.91) [38]. Cronbach’s alpha for the current sample was 0.92.
2. Beck Scale for Suicidal Ideation (BSS) [39]. Twenty one items that measure suicidal thoughts and behaviours. Good test-retest reliability (r = 0.88) [40] and good internal reliability in clinical samples (α = 0.84) [41] Cronbach’s alpha for the current sample was 0.97.
3. Beck Hopelessness Scale (BHS) [34]. Twenty items that measure pessimistic beliefs about the future with good test-retest reliability (r = 0.85) [42] and good to excellent internal reliability in clinical samples (Kuder-Richardson reliabilities ranging from 0.87 to 0.93) [34]. Cronbach’s alpha for the current sample was 0.92.
4. The Defeat scale [35]. Sixteen items assessing an individual’s failed struggle, powerlessness and perceived low social status, which has excellent internal consistency in clinical samples (α = 0.93) [35]. Cronbach’s alpha for the current sample was 0.95.
5. The Entrapment scale [35]. Sixteen items assessing an individual’s feeling of being trapped and wishing to escape, which has good internal consistency in clinical samples (α = 0.86) [35]. Cronbach’s alpha for the current sample was 0.94.
6. Metacognitions questionnaire-30 (MCQ-30) [36]. Thirty items which assess metacognition. Wells and Cartwright-Hatton (2004) reported α ranging from 0.72–0.93 for the various subscales [36]. The five subscales of the MCQ-30 and corresponding α for the current sample are: (i) positive beliefs about worry (0.79); (ii) negative beliefs concerning uncontrollability and danger (0.81); (iii) cognitive confidence (0.89); (iv) negative beliefs concerning the consequences of not controlling thoughts (0.69); (v) cognitive self-consciousness (0.86).

The aims of the current study were to investigate the role of metacognitive beliefs so the MCQ-30 subscale of cognitive self-consciousness was not included in the analysis of subscale scores.

### 2.3. Procedure

Referrers provided written information to those who met the study criteria. Interested individuals consented to be contacted and were then approached by the researchers who took written informed consent. Demographic details were taken alongside the above self-report measures (the order of completion was randomised to prevent systematic bias). The participants accessed the online ESM diary items via a web-link embedded within a text message sent to their mobile telephone. This began within 24 h of completing the self-report questionnaires. The web-link to the diary items closed within 30 min of a participant receiving a text message meaning systematic bias due to delayed response could be reduced. Participants were prompted to complete the ESM diary for 6 consecutive days, 7 times per day at pseudo-random intervals. Entries were completed, on average, every two hours between the hours of 08:00 and 22:00. The range of the number of completed diary entries for participants was between 1 and 40 with 42 entries being the maximum.

### 2.4. Statistical Analysis

The design of the study allowed for both cross-sectional and micro-longitudinal analyses (across 7 days) to provide a more detailed examination of the relationships between metacognitive beliefs and suicidal ideation. Descriptive and correlational analysis of the trait level self-report questionnaires was completed using SPSS version 22.0 for Windows (IBM SPSS Statistics for Windows, Version 22.0. Armonk, NY: IBM Corp.). Non-parametric analysis (Spearman’s correlation co-efficient) was performed for trait-level data, due to the non-normal distribution of suicidal ideation scores. Data from participants who completed at least one diary entry were included in the multi-level modelling analysis. Although some have suggested a minimum number of diary entries to be included [43], there is no theoretical basis to this. A sensitivity analysis was conducted finding very similar results when all participants were included in the analysis compared to only those with a minimum number of completed entries included. To examine the effect of the state-level predictor variables on the state-level outcome variables, a multilevel modelling approach, estimated by maximum likelihood, was used. Multilevel modelling is required for the analysis of ESM data, since the data consists of multiple observations within a participant (diary item responses nested within days nested within participants) and so each observation is not independent. Multilevel models also estimate the variability associated within the three levels of data, and so allows for the random variation between participants to change over time. Each state level predictor was entered into a multilevel model individually and only those predictors that were significantly associated with scores of suicidal ideation were then selected for entry into the multivariate model. Both participant and day were entered as random effects in models. *p* values less than or equal to 0.05 were considered significant. Multilevel modelling was undertaken with STATA Intercooled software version 14.0 [44].

Across all analyses, missing data were found to be missing at random. Due to the impact of missing data on total scores for trait level measures, data were imputed using expectation maximisation. For the state-level diary items, an average score was taken from across the two item scores on each variable, meaning that the number of data points for each hypothesis varied slightly.

A separate analysis of this ESM dataset, focused upon the roles of rumination and thought control strategies, has previously been reported [45].

## 3. Results

### 3.1. Descriptive Statistics

Twenty-seven participants completed the trait level questionnaire measures. Table 3 presents general descriptive statistics and a correlation matrix of the questionnaires.

Correlation analyses of trait level self-report measures found that depression, hopelessness, defeat and entrapment were all significantly and positively correlated with suicidal ideation (see Table 3). No significant correlations were found for trait-level measures of metacognition and suicidal ideation. Scores of suicidal ideation were higher than other samples, indicating that the current sample were experiencing significant suicidal distress. Dervic et al. (2006) found depressed participants average scores on the BSS were 11.5 (SD = 10.3) [46]. Knott and Range (2001) also had a lower average on the BSS with participants from a community mental health team [47].

### 3.2. Diary Protocol

Of the 27 participants, 24 (89%) completed at least one state level diary entry. Four hundred and ninety-three diary entries (each entry represents a single time point) were completed resulting in an average of 21 entries per participant (average compliance rate of 49%). This is lower than in samples reported in a review evaluating the use of ESM in suicidal participants with compliance rates ranging from 58% to 86% [29] and in a separate study exploring affect variability in suicide reporting a compliance rate of 58% [43]. These studies mainly recruited individuals with lower levels of suicidal ideation than those observed in the current sample, which could account for this difference in compliance. Within the current sample, there were no group differences in the number of completed diary entries between community and in-patient participants (t = 1.38, *p* = 0.18), between those who had previously attempted suicide and those who had no such history (t = −0.18, *p* = 0.86), and between males and females (t = 0.91, *p* = 0.38).

**Hypothesis** **1.** *ESM state-level measures of depression, hopelessness, defeat entrapment and cognitive confidence were statistically significantly correlated with the equivalent trait-level self-report questionnaire as demonstrated in Table 4. The relationship was not significant for the measures of positive and negative metacognition and the need to control thoughts.*

**Hypothesis** **2.** *ESM state-level scores for negative affect (β = 0.477, p < 0.001), hopelessness (β = 0.204, p < 0.001) and defeat (β = 0.280, p < 0.001) remained significantly and independently associated with suicidal ideation when other variables were controlled for. Whilst entrapment was significantly associated with suicidal ideation in a univariate model (β = 0.531, p < 0.001), entrapment was not a significant predictor in the multivariate model (β = 0.023, p = 0.651).*

**Hypothesis** **3.** *Cognitive confidence (β = 0.309, p < 0.001), suicide-specific positive metacognitive beliefs (β = 0.567, p < 0.001) and suicide-specific negative metacognitive beliefs (β = 0.621, p < 0.001) were all found to be significantly associated with suicidal ideation at a univariate level, and thus entered into the multivariate model. Only positive (β = 0.468, p < 0.001) and negative (β = 0.504, p < 0.001) metacognitive beliefs about suicidal thinking were independently and significantly associated with suicidal ideation in the multivariate model.*

**Hypothesis** **4.** *The metacognition variables that were found to be significantly associated with suicidal ideation (positive and negative metacognitive beliefs about suicidal ideation) were independently entered into a model for suicidal ideation, whilst also controlling for negative affect, hopelessness and defeat (see Table 5). Entrapment was not found to be significantly associated with suicidal ideation and so it was not included in the model. Positive (β = 0.241,* *p < 0.001) and negative (β = 0.167,* *p < 0.001) metacognitive beliefs about suicidal thinking remained significantly associated with suicidal ideation when negative affect, hopelessness and defeat were controlled for. The metacognitive variables of positive and negative metacognitive beliefs about suicidal ideation made statistically significant contributions to the prediction of suicidal ideation, over and above the contributions made by established cognitive variables.*

## 4. Discussion

This study was the first to examine metacognitive beliefs in relation to suicidal ideation, using experience sampling methodology. A multilevel model examined whether metacognitive beliefs about suicidal ideation were significantly associated with suicidal ideation when known cognitive correlates of suicide were accounted for. All hypotheses were fully or partially supported. Positive and negative metacognitive beliefs about suicidal ideation were significantly associated with state-level scores of suicidal ideation when cognitive correlates were controlled for. This further validates the S-REF model [6,7]. Suicidal ideation might therefore be viewed as a perseverative cognitive process and part of the CAS in a similar way that worry and rumination are conceptualised in anxiety and depression.

State-level measures of depression, defeat, entrapment, hopelessness and cognitive confidence were significantly correlated with established trait-level measures, thus providing validation of the state-level measurement. This was not the case for positive and negative metacognitive beliefs and beliefs about the need to control thoughts with no significant correlation between the trait and state measures. At the trait level, the MCQ-30 was used as a proxy measure of metacognitive beliefs about suicidal ideation due to the absence of any current measure of metacognitive beliefs about suicidal ideation. The lack of a significant correlation is understood in the context that metacognitions measured in the MCQ-30 and metacognitive beliefs about suicidal ideation are subtly different constructs. No significant relationships were observed at the trait level between the MCQ-30 subscales and suicidal ideation although the sample size (*n* = 27) means this analysis was likely underpowered to detect a significant result. Future studies would need to refine the measurement of metacognitive beliefs about suicidal ideation at both the state and trait-level.

Evidence of the association between known cognitive correlates of suicide was also provided. Although entrapment was significantly associated with suicidal ideation in a univariate model, when entered into a multivariate model, which also included the contributions of negative affect, hopelessness and defeat, there was no longer a significant association. Others have suggested that defeat and entrapment are best defined as a single construct [48], which could account for this finding.

ESM is a relatively novel methodology that allows for an ecologically valid examination of relationships between variables. Multilevel modelling is required, which has been argued to be more precise [49] given its ability to account for variables that are not independent. The combination of greater ecological validity with more precise statistical analysis allows for a greater confidence in these results.

The results demonstrate that thinking of suicide is appraised as both a coping strategy and a process that is uncontrollable and harmful. Based on this evidence and combined with potential future research developing a theoretical account of this relationship, a specific metacognitive treatment could be developed. Metacognitive treatment has demonstrated considerable efficacy for other psychological disorders [8,50] and so could potentially offer a life-saving treatment for those experiencing suicidal ideation.

Prior to this, it would be important to further understand positive and negative metacognitive beliefs held about suicidal ideation. Qualitative research could offer this and contribute to the development of a measure of metacognitions specific to suicidal ideation. A more valid and reliable measure would allow for greater accuracy for both research and evaluation of any interventions. Items measuring suicidal ideation were limited to two items and so further work examining the relationship with more specific elements of ideation (e.g., planning and intention) is also needed.

It would also be important to develop an understanding of the causal mechanisms and processes linking metacognitive beliefs, other metacognitive processes and the transition from suicidal ideation to behaviour. Williams, Duggan, Crane and Hepburn (2011) demonstrated that there is a positive association between suppression of suicidal ideation and perceived severity of historical suicidal ideation [22]. Hallard, Wells, Aadahl, Emsley and Pratt (2021) have explored the contribution of rumination and thought control strategies in relation to suicidal ideation [45]. Maladaptive thought control strategy use (worry and punishment), alongside rumination, was found to predict suicidal ideation. Adaptive strategies (distraction, social control and reappraisal) emerged as negative predictors. Future research should consider other processes as predicted by the S-REF model on suicidal ideation to generate a complete metacognitive model of suicidal ideation. Klonsky and May (2015) argued that any robust model of suicide needs to explain mechanisms leading to both suicidal ideation and suicide behavior [4]. The contribution of metacognitive beliefs to suicidal behaviour is currently unknown. It is important the theoretical understanding of the specific role of the S-REF model to both suicidal ideation and behaviour is developed prior to any clinical interventions.

### Limitations

Although many data points were obtained, there was a limited range of characteristics reported by participants, for example, all participants self-identified as being white and a majority as female, thus affecting generalisability. The compliance rate of 49% is lower than found in other studies. It would be important to replicate these findings in a broader range of participants. The sample size also means that caution should be observed with any conclusions drawn from the correlation analyses of trait-level measures, although these data were presented primarily for descriptive purposes.

## 5. Conclusions

Positive and negative metacognitive beliefs about suicidal ideation are significantly associated with suicidal ideation when known correlates of suicidal ideation are controlled for. This supports the S-REF model [6,7] and presents a need to further extend existing cognitive models of suicide. Such models may benefit from considering suicide-specific metacognitive beliefs that are contributing to, and possibly maintaining, suicidal ideation, and the potential influence of other metacognitive processes. Further research is required to establish a better understanding of the metacognitive mechanisms involved in suicidal ideation with the eventual aim of developing more effective interventions.

## Figures and Tables

**Table 1:** Sample demographics.

Demographic	Frequency	%
Site: - In-patient - Community	20 7	74 26
Gender: - Female - Male	18 9	66 34
Ethnicity (self-report): - White British - White Other	25 2	93 7
Primary diagnosis (self-report): - Personality disorder - Affective/Mood disorder - Psychotic disorder - Eating disorder - Not stated	7 12 2 1 5	26 45 7 3 19
Lifetime history of suicide attempt (self-report): - Yes - No - Not stated	21 3 3	78 11 11
Highest qualification: - Higher education (e.g., College, University) - A-level - GCSE - No Qualification - Not stated	10 4 4 5 4	36 15 15 19 15

**Table 2:** ESM diary items and related construct.

Diary Instruction	Diary Item	Construct
Right now…	I want to die	Suicidal ideation
	I feel unhappy	Negative affect
	I feel anxious	Negative affect
	I feel powerless	Defeat
Just before the text…	I was thinking about killing myself	Suicidal ideation
Right now, how much do you agree with the following…	It is bad to have thoughts of killing myself	MCB *: Need to control thoughts
	My suicidal thoughts persist, no matter how I try to stop them	Negative MCB: Uncontrollability
	I have no control over my suicidal thoughts	Negative MCB: Uncontrollability
	Thinking about suicide is dangerous for me	Negative MCB: Harm
	If I don’t stop my suicidal thoughts I will go mad	Negative MCB: Harm
	Thinking about suicide helps me cope	Positive MCB
	Thinking of ending it all gives me peace of mind	Positive MCB
	I have a poor memory	Cognitive Confidence
	I think a lot about my suicidal thoughts	Cognitive Self-consciousness
	I am constantly aware of my suicidal thoughts	Cognitive Self-consciousness
	I look forward to the future	Hopelessness
	Things don’t work out the way I want	Hopelessness
	I am one of life’s losers	Defeat
	I am trapped in my situation	Entrapment
	There are things in my life I want to escape	Entrapment

* MCB = suicide-specific metacognitive beliefs.

**Table 3:** Descriptive statistics and Spearman’s correlation coefficients for trait level self-report questionnaire measures (*n* = 27).

Self-Report Measure	Mean	SD	Range	1.	2.	3.	4.	5.	6.	7.	8.
1. Suicidal ideation (BSS)	20.98	11.89	38								
2. Depression (BDI)	37.24	13.01	53	0.57 **							
3. Hopelessness (BHS)	15.22	5.22	19	0.58 **	0.70 ***						
4. Defeat (Defeat scale)	46.78	14.00	57	0.54 **	0.93 ***	0.66 ***					
5. Entrapment (Entrapment scale)	41.93	15.17	62	0.48 *	0.76 ***	0.61 **	0.81 ***				
Metacognitions (MCQ-30):											
6. Cognitive confidence	14.23	5.31	17	0.01	0.30	0.04	0.32	0.32			
7. Positive metacognitive beliefs	9.91	3.43	12	−0.07	0.07	0.11	0.04	0.21	−0.15		
8. Negative metacognitive beliefs	17.67	4.31	17	−0.06	0.34	−0.02	0.26	0.21	0.16	−0.25	
9. Need to control thoughts	14.78	4.19	8	0.29	0.34	0.06	0.25	0.34	0.07	−0.00	0.57 **

Note: BSS = Beck Scale of Suicidal Ideation; BDI = Beck Depression Inventory; BHS = Beck Hopelessness Scale; MCQ-30 = Metacognitions Questionnaire-30. *** *p* < 0.001 ** *p* < 0.01 * *p* < 0.05 (2 tailed).

**Table 4:** Spearman’s correlation co-efficients for associations between trait and state level measures.

Cognitive Construct	Correlation Co-Efficient	Meta-Cognitive Construct	Correlation Co-Efficient
Suicidal ideation	0.71 ***	Cognitive confidence	0.61 ***
Depression	0.70 ***	Positive metacognitive beliefs about suicidal ideation	0.20
Hopelessness	0.80 ***	Negative metacognitive beliefs about suicidal ideation	0.22
Defeat	0.79 ***	Need to control thoughts	0.23
Entrapment	0.74 ***		

*** *p* < 0.001 (1 tailed).

**Table 5:** Concurrent associations between positive and negative metacognitive beliefs about suicide and suicidal ideation (dependent variable) when all variables entered simultaneously and the effects of negative affect, hopelessness and defeat were controlled for.

Model 1					
				CI	
**Metacognitive Variables**	β	SE	*p*	Lower	Upper
Positive metacognitive beliefs about suicidal ideation	0.241	0.041	<0.001	0.160	0.323
Negative metacognitive beliefs about suicidal ideation	0.167	0.045	<0.001	0.079	0.255
**Cognitive Variables**					
Negative affect	0.417	0.040	<0.001	0.339	0.494
Hopelessness	0.176	0.047	<0.001	0.083	0.269
Defeat	0.208	0.049	<0.001	0.111	0.305

## Data Availability

The data presented in this study are available on request from the corresponding author. The data are not publicly available due to their containing information that could compromise the privacy of research participants.

## Supplementary Materials

The following are available online at https://www.mdpi.com/article/10.3390/ijerph182312336/s1, Table S1: Full list of all ESM diary items and related construct.
