# Supplementary material for: Metacognitive Beliefs and Suicidal Ideation: An Experience Sampling Study
Source: Int J Environ Res Public Health. 2021 Nov 24;18(23):12336. doi: 10.3390/ijerph182312336 (PMC8656543; doi:10.3390/ijerph182312336)
Supplement: Supplementary file 1 [file ijerph-18-12336-s001.zip › ijerph-1430229-supplementary.pdf]

*Article:* ***Metacognitive beliefs and suicidal ideation: An experience sampling study***

**Supplementary Materials:**

**Table S1.** Full list of all ESM diary items and related construct.

| Diary instruction | Diary item | Construct |
|---|---|---|
| Right now… | I want to die | Suicidal ideation |
| | I feel unhappy | Negative affect |
| | I feel anxious | Negative affect |
| | I feel powerless | Defeat |
| | | |
| Just before the text… | I was thinking about killing myself | Suicidal ideation |
| | I was thinking about my failures and weaknesses | Rumination |
| | I was analysing why I feel the way I do | Rumination |
| | I was worrying about what goes on in my head | Meta-worry |
| | I was worrying about the amount I worry | Meta-worry |
| | | |
| Right now, how much do you agree with the following… | It is bad to have thoughts of killing myself | MCB*: Need to control thoughts |
| | My suicidal thoughts persist, no matter how I try to stop them | Negative MCB: Uncontrollability |
| | I have no control over my suicidal thoughts | Negative MCB: Uncontrollability |
| | Thinking about suicide is dangerous for me | Negative MCB: Harm |
| | If I don't stop my suicidal thoughts I will go mad | Negative MCB: Harm |
| | Thinking about suicide helps me cope | Positive MCB |
| | Thinking of ending it all gives me peace of mind | Positive MCB |
| | I have a poor memory | Cognitive Confidence |
| | I think a lot about my suicidal thoughts | Cognitive Self-consciousness |
| | I am constantly aware of my suicidal thoughts | Cognitive Self-consciousness |
| | I look forward to the future | Hopelessness |
| | Things don't work out the way I want | Hopelessness |
| | I am one of life's losers | Defeat |
| | I am trapped in my situation | Entrapment |
| | There are things in my life I want to escape | Entrapment |

*Article:* ***Metacognitive beliefs and suicidal ideation: An experience sampling study***

| | | |
|---|---|---|
| Since the last text, I have been responding to my thoughts by… | Dwelling on other worries | TCS*: worry |
| | Focussing on other negative thoughts | TCS: worry |
| | Getting angry at myself | TCS: punishment |
| | Telling myself that something bad will happen if I think those thoughts | TCS: punishment |
| | Doing something I enjoy | TCS: distraction |
| | Keeping myself busy | TCS: distraction |
| | Talking to a friend about the thought | TCS: social |
| | Asking for reassurance | TCS: social |
| | Analysing my thoughts, rationally | TCS: reappraisal |
| | Thinking about things in a different way | TCS: reappraisal |
| | Thinking I would be better off ending it all | TCS: suicidal ideation |

*MCB = suicide-specific metacognitive beliefs
*TCS = thought control strategy

Response to items in blue were not analysed within the current study. These items were analysed in the study reported by Hallard, R., Wells, A., Aadahl, V., Emsley, R., & Pratt, D. (2021). Metacognition, rumination and suicidal ideation. An experience sampling test of the self-regulatory executive function model. *Psychiatry Research, 303*, 114083.
